# Metastatic Pancreatic-Biliary Cancer Presenting as Intramuscular Fluid Collections: A Case Report

**DOI:** 10.7759/cureus.8943

**Published:** 2020-07-01

**Authors:** Romil Singh, Tony Oliver, Uma Motapothula, Sawai Singh Rathore, Dhwani Kamrai

**Affiliations:** 1 Internal Medicine, Metropolitan Hospital, Jaipur, IND; 2 Internal Medicine, University of South Dakota Sanford Medical Center, Sioux Falls, USA; 3 Internal Medicine, Sanford Health, Sioux Falls, USA; 4 Internal Medicine, Dr. Sampurnanand Medical College, Jodhpur, IND; 5 Psychiatry, Griffin Memorial Hospital, Norman, USA

**Keywords:** pancreatic-biliary cancer, intramuscular fluid, cancer patients, diarrhea, pancreato-biliary, mri, usg

## Abstract

At a certain juncture, when clinicians are unable to gather information about a particular disease especially due to patient's unobtrusive findings, the presence of an aberrant connection might yield a diagnosis. Herein we present the findings of an unusual enlightening case of an 81-year-old Caucasian male with a history of bowel resection, poor appetite, generalized weakness, ptosis, and two weeks of weight loss. The computerized tomography scan revealed several sets of an abdominal intramuscular fluid collection with enhancements in the surrounding, indicative of several abscesses, and brain and spine magnetic resonance imaging indicated right-orbital metastasis in the superior rectal muscles. A biopsy of the cystic lesion of the anterior abdominal wall revealed poorly differentiated metastatic adenocarcinoma, most consistent with the primary pancreaticobiliary origin. This case report sums up this innovative portrayal of metastatic cancer as an intramuscular fluid collection.

## Introduction

An estimated 57,600 adults, consisting of 30,400 males and 27,200 females, are predicted to be diagnosed in 2020 with pancreatic carcinoma in the United States (US) [1, 2]. The disorder is responsible for around 3% of all cancer cases. Incidence rates of African-American people are 25% higher than in the Caucasian population. Pancreatic cancer is the ninth most common malignancy in females and the tenth most common in males. It is the fourth leading cause of cancer-related death in both males and females. It accounts for 7% of all cancer-related deaths, of which 93% happens to be of exocrine adenocarcinoma pancreatic origin [2].

Pancreatic cancer patients are estimated to have a five-year survival rate of 9% [1,2]. Amongst the small number of people diagnosed with local disease (10%), the five-year survival rate is 37%. Most patients are diagnosed at a distant stage (53%) with a 3% five-year survival period [2]. Pancreato-biliary carcinoma patients present with obstructive jaundice caused by tumor obstruction of the distal bile duct in about 80% of the cases. Ampullary cancers are not typically regarded as a source of obstructive jaundice due to their lower incidence rates compared to other periampullary malignancies [1].

Transabdominal ultrasound (US) is an appropriate first test in patients with obstructive jaundice, but typically it would not display the tumor. Helical computed tomography (CT) scanning for visualizing the pancreas and associated structures is usually required. Although its spatial resolution is inadequate to assess the degree of local tumor invasion, it is the most effective tool to rule out the existence of distant metastases [1]. Endoscopic retrograde cholangiopancreatography (ERCP) is a valuable endoscopic analysis as it enables tumor decompression if required. Endoscopic ultrasonography (EUS) is sensitive in detecting small ampullary tumors. EUS is superior to CT and the transabdominal US; it is not typically required for diagnosis. Consequently, EUS is not widely used for the treatment and staging of ampullary carcinoma [1].

## Case presentation

**Figure 1:**
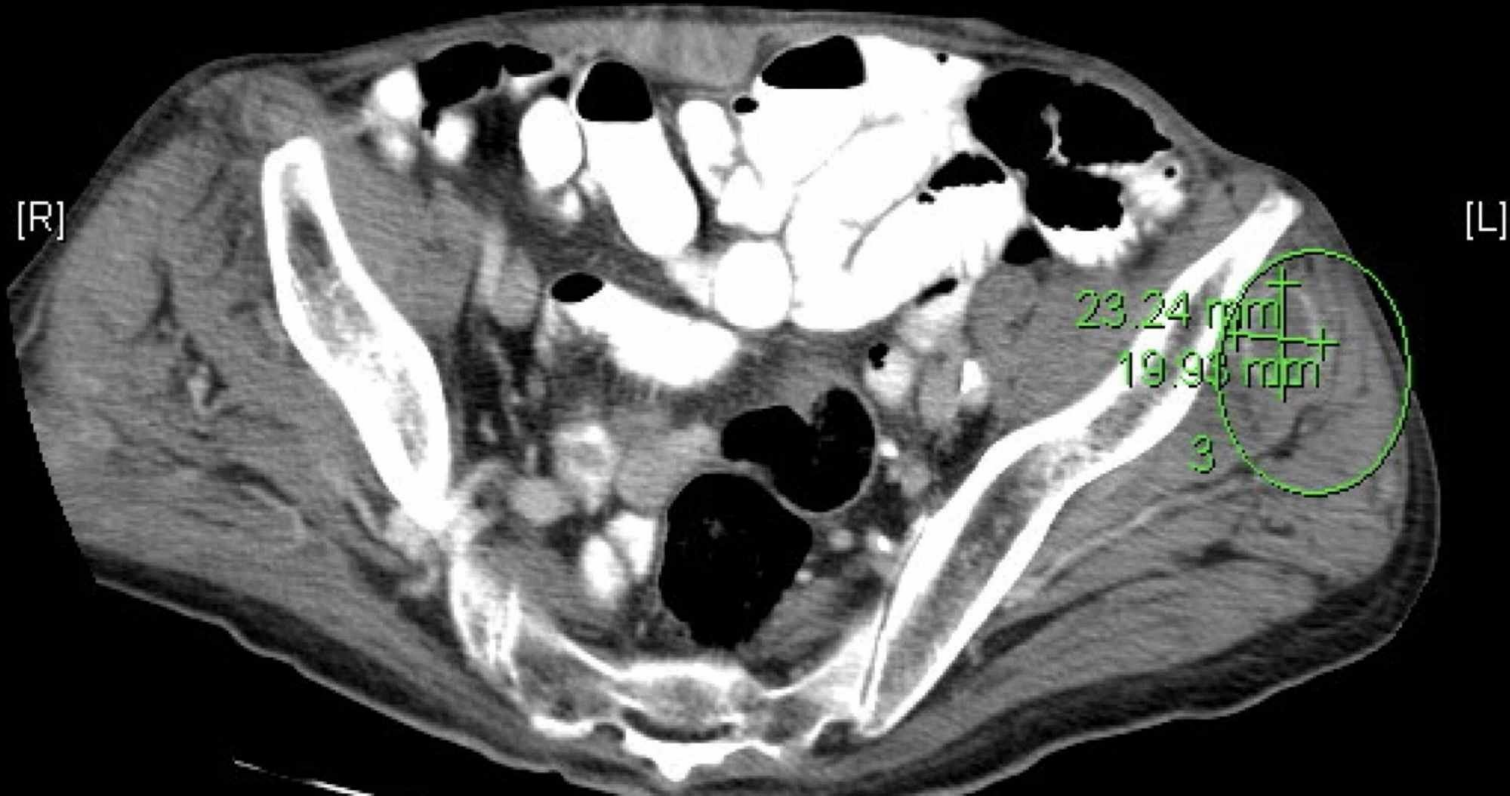
Abdominal computerized tomography scan of the patient showing intramuscular fluid collections with surrounding enhancement, indicating of abscesses

An 81-year-old Caucasian male with a past surgical history of bowel resection (post road traffic accident at a very young age) was admitted with poor appetite, generalized weakness, and weight loss for two weeks. The patient had 30 pack-years of smoking history, however, he denied alcohol use. On examination, he was cachectic and found to have right eye ptosis and multiple non-tender, non-erythematous lumps of about 2 cm in diameter on the left arm, left scapular area, and abdominal wall. His vitals were 98.5 F/36.9 C (temperature), blood pressure was 118/64 mm Hg, and 94/min pulse rate. Respiratory rate was 16/min, and oxygen (O2) saturation was 94% on room air. At the time of admission, laboratory data showed elevated white blood cells (WBC) of 19,000, hemoglobin (Hb) 12.6, platelet count of 708, and a bilirubin level of 10 mg/dl. Auscultation revealed normal respiratory and heart sounds. The abdomen was soft non-tender, with normal bowel sounds. A lump was found in the right periumbilical area with multiple small lumps in the left lower quadrant and bilateral inguinal adenopathy. The neurological evaluation revealed right-sided ptosis with normal power, sensations, and reflexes. CT scan of the lung showed medial right lower lobe consolidation. He was started on piperacillin-tazobactam for suspected abscess. An abdominal computerized tomography scan of the patient showed intramuscular fluid collections with surrounding enhancement indicating of abscesses (Figure 1).

A magnetic resonance imaging (MRI) of the brain and spine showed right retro-orbital metastasis likely arising from the superior rectus muscle. A cervical and thoracic spine MRI showed widespread bone metastasis with pathologic fractures of the thoracic ninth (T9) and thoracic twelfth (T12) vertebral bodies. He underwent a biopsy of cystic lesion in the abdomen which on histopathology, reported as adenocarcinoma. Further, on performing immunohistochemistry (IHC), tumor cells were positive for CK7 and CK19 and tested negative for CDX2, favoring the diagnosis of metastatic adenocarcinoma of pancreaticobiliary origin.

An oncologist continued to follow up with the patient in an outpatient setting regarding his treatment for the metastatic pancreaticobiliary adenocarcinoma. It was later discovered that the patient refused any further treatment and preferred to be managed by the primary care physician (PCP). Unfortunately, he passed away soon after the diagnosis.

## Discussion

Pancreaticobiliary cancer consists of cancers of the ampullary, peri-ampullary, and bile duct. In our case, the patient had a sporadic presentation of underlying adenocarcinoma originating from the pancreaticobiliary region. Diagnosing pancreatic cancer in a patient without symptoms is complex. It is due to the lack of specific, cost-effective screening tests that could find early-stage pancreatic cancer efficiently, reliably, and accurately in individuals without symptoms. Delay in diagnosis often causes cancer infiltration to the surrounding structures and distant metastasis. Thus, making them a poor surgical-resection candidate [2].

Periampullary tumors are neoplasms that occur in the vicinity of Vater's ampulla. The neoplasms that arise at this site can stem from the pancreas, duodenum, distal common bile duct (CBD), or ampullary complex. Ampullary carcinomas are classified in the ampullary complex as those that are distal to the juxtaposition of the pancreatic duct and the distal common bile duct. Due to their lower incidence relative to other periampullary malignancies, ampullary cancers are not usually suspected as a cause of obstructive jaundice [1,2].

Typical pancreaticobiliary cancer nonspecific signs include diarrhea induced by fat malabsorption (steatorrhea), fatigue, and mild weight loss [1]. Our patient presented with similar findings at the time of his admission. The nodal involvement is associated with a relatively much worse prognosis [2].

The symptom of jaundice is found in about 80% of the ampullary cancer patients caused due to tumor obstruction of the distal bile duct [1]. A combination of endoscopic, radiological, and histological features help to achieve diagnosis and staging. The primary two considerations are tumor identification and distinction from an ampullary adenoma or tumor that arises outside the ampulla, usually in the form of pancreatic carcinoma or distal cholangiocarcinoma [3].

The prognosis of an ampullary carcinoma in the absence of metastases depends primarily on two factors: the extent of local tumor invasion, as indicated by the T stage, and the prevalence of lymphatic spread, as expressed by the N stage. The tumor, node, metastasis (TNM) method of the American Joint Committee on Cancer (AJCC) and Union for International Cancer Control (UICC) is the most commonly used method for tumor classification. The latest eighth edition of the 2017 version includes a variety of reclassifications resulting in improved prognostic and clinical relevance compared to the previous edition. It includes revised interpretations of the traditional TNM staging as well as the introduction of various subsets [4]. Our patient falls in the N2 category.

**Figure 2:**
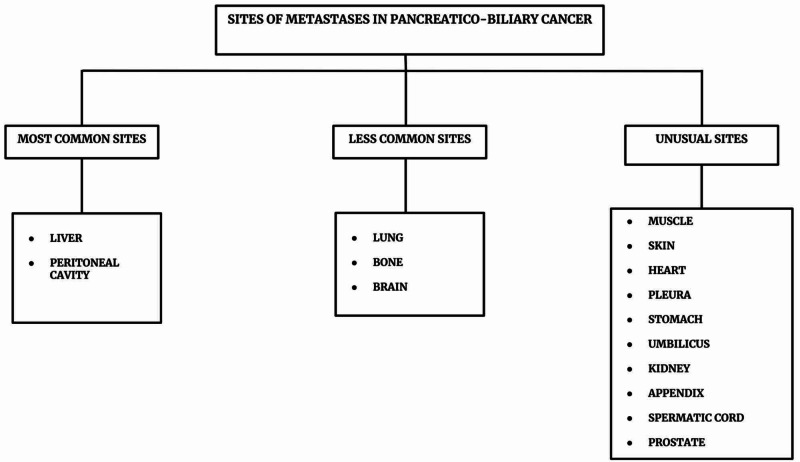
Metastatic sites of pancreaticobiliary cancer Illustration of the common and uncommon sites of metastases of pancreaticobiliary cancer [6].

Metastases to the abdominal wall can occur via numerous routes, including its ample arterial supply, lymphatic system, or veno-systemic circulation. The metastases are generally more prominent around the umbilical region. Besides, the literature has described umbilical invasion through embryonic residue, i.e., umbilical ligament. Also, abdominal wall metastasis can occur via direct peritoneal extension and postoperative seeding of the abdominal wall [5]. Common and uncommon metastatic sites of pancreaticobiliary cancer are illustrated in Figure 2 [6].

Plaza et al. reported that most soft tissue metastases are poorly differentiated and show signs of advanced disease in a study conducted over 30 years in a large academic medical center. About 27% of patients presented with soft tissue metastasis as an initial manifestation, but none of these 32 patients out of 118 had pancreatic cancer [7]. In another study, Surov et al. reported that in over 481 patients, 682 muscle metastases were observed, 21% of which were gastrointestinal, but none of which had primary tumor origin from the pancreas [8]. Chisthi and Manju reported metastasis from pancreatic cancer to skeletal muscles such as calf muscles [9]. Abdominal wall metastases are described in the literature primarily in postoperative cases. Studies in the past have described cutaneous metastases from pancreatic cancer, citing umbilicus as the most common site for cutaneous metastases [10,11]. In our case, rather than a solid mass, the biopsy was taken from a cystic lesion. Therefore, we suggest that in patients with inconspicuous findings, the abdominal wall cyst assessment can provide a clue about the nature of the lesion and its origin. In our patient, the clinical examination established a malignant lesion, primarily of adeno-carcinomatous origin from the pancreaticobiliary tract.

## Conclusions

Addressing the complexities of pancreaticobiliary cancer is an arduous job, especially when the findings are atypical. Results found in this case are unique in the sense that a novel metastasis presentation was occurring in the anterior abdominal wall rather than metastasis to the liver and lymph nodes. Our findings suggest that using an abdominal cyst lesion examination in such a presentation might provide us a piece of evidence and information about the nature of the lesion, its origin, and also assist in management. Clinicians can use this unconventional finding to have an improved understanding of this varied presentation of pancreaticobiliary cancer.
